# Identifying sources of antibiotic resistance genes in the environment using the microbial *Find, Inform*, and *Test* framework

**DOI:** 10.3389/fmicb.2023.1223876

**Published:** 2023-09-05

**Authors:** Corinne Wiesner-Friedman, Rachelle E. Beattie, Jill R. Stewart, Krassimira R. Hristova, Marc L. Serre

**Affiliations:** ^1^Office of Research and Development, U.S. Environmental Protection Agency, Cincinnati, OH, United States; ^2^U.S. Geological Survey, Columbia Environmental Research Center, Columbia, MO, United States; ^3^Department of Biological Sciences, Marquette University, Milwaukee, WI, United States; ^4^Gillings School of Global Public Health, Department of Environmental Sciences and Engineering, University of North Carolina at Chapel Hill, Chapel Hill, NC, United States

**Keywords:** microbial FIT, antimicrobial resistance, surface water, sediment, animal feeding operations, land application

## Abstract

**Introduction:**

Antimicrobial resistance (AMR) is an increasing public health concern for humans, animals, and the environment. However, the contributions of spatially distributed sources of AMR in the environment are not well defined.

**Methods:**

To identify the sources of environmental AMR, the novel microbial Find, Inform, and Test (FIT) model was applied to a panel of five antibiotic resistance-associated genes (ARGs), namely, erm(B), tet(W), qnrA, sul1, and intI1, quantified from riverbed sediment and surface water from a mixed-use region.

**Results:**

A one standard deviation increase in the modeled contributions of elevated AMR from bovine sources or land-applied waste sources [land application of biosolids, sludge, and industrial wastewater (i.e., food processing) and domestic (i.e., municipal and septage)] was associated with 34–80% and 33–77% increases in the relative abundances of the ARGs in riverbed sediment and surface water, respectively. Sources influenced environmental AMR at overland distances of up to 13 km.

**Discussion:**

Our study corroborates previous evidence of offsite migration of microbial pollution from bovine sources and newly suggests offsite migration from land-applied waste. With FIT, we estimated the distance-based influence range overland and downstream around sources to model the impact these sources may have on AMR at unsampled sites. This modeling supports targeted monitoring of AMR from sources for future exposure and risk mitigation efforts.

## 1. Introduction

Antimicrobial resistance (AMR) currently exists at higher than natural levels due to antibiotics use and misuse in human and animal medicine and livestock production (Davies and Davies, 2010). Wastes from these origins contain pathogens and associated antibiotic resistance genes (ARGs), which can be dispersed *via* runoff into rivers in the environment (Amarasiri et al., 2019). While putative geographical sources of pathogens and ARGs have been identified, the extent of their contributions to elevated AMR in the environment remains unknown. Characterizations of AMR spatial sources, their contributions (Nappier et al., 2020), and modeling approaches that suit the conceptual framework of ARG transport, attenuation, and amplification (Singer et al., 2006; Pruden et al., 2012) are needed.

Antibiotic resistance genes and antibiotic-resistant bacteria (ARB) can become elevated intracellularly due to the impacts of human and animal sources by (1) direct dissemination or extracellularly and through horizontal gene transfer (HGT), (2) organic matter enrichment of ARGs, and (3) the dissemination of selective factors (e.g., antibiotics) from sources (Xie et al., 2018). A geospatial study of surface water in the United States supports that land use is a driver of AMR (Keely et al., 2022). Potential sources of elevated AMR in the environment have been identified at locations where human, animal, and industrial waste meet with the environment (Nappier et al., 2020; Zainab et al., 2020; Zheng et al., 2021). Animal feeding operations (AFOs) are likely sources due to frequent antibiotic use for disease treatment and prevention or use in animal feed, often at sub-therapeutic doses (Pruden et al., 2012; Ling et al., 2013; Heaney et al., 2015; Li et al., 2015; Rogers et al., 2018; Lopatto et al., 2019). Other potential sources include wastewater treatment plants (WWTPs) (Bueno et al., 2018; Brown et al., 2019; Pazda et al., 2019; Beattie et al., 2020b) and land application sites of treated (i.e., biosolids and semi-solids) and untreated wastes (i.e., biosolids and wastewater) from agricultural (e.g., manure spreading), industrial, or municipal origins (Munir and Xagoraraki, 2011; Beattie et al., 2018, 2020b; Pepper et al., 2018; Yang et al., 2018; Duarte et al., 2019; Jacobs et al., 2019). Additionally, elevated AMR levels have been detected in groundwater near septic systems (O'Dwyer et al., 2017), and low-intensity developed land cover is a fecal contamination source (Crowther et al., 2003; Alford et al., 2016; Bucci et al., 2017; Hinojosa et al., 2020; McKee et al., 2020; Wiesner-Friedman et al., 2021a). However, ARGs predate manufactured antimicrobials and exist in natural environments (D'Costa et al., 2011; Van Goethem et al., 2018), and different soil types can represent sources (Zhang et al., 2018, 2021; Macedo et al., 2020). Additionally, season (Beattie et al., 2018, 2020a; Zheng et al., 2018; Liu et al., 2020) and precipitation (Ahmed et al., 2018; Keen et al., 2018) are temporal factors to consider.

To model contributions from these sources with known mechanistic approaches (Wang et al., 2019; Costa et al., 2021), knowledge of loadings and decay is needed. While current research shows that first-order decay can represent ARG levels over distance and time and that ARG decay occurs over long time scales (e.g., weeks to months), the decay rate varies depending on the ARG and environmental variables (Mao et al., 2014; Lopatto et al., 2019; Macedo et al., 2020; Barrios et al., 2021; Burch et al., 2021). Furthermore, loadings at sources are not well characterized. Statistical models incorporating first-order decay are helpful to screen potential sources of elevated AMR without requiring mechanistic information (Wang et al., 2019; Costa et al., 2021). Here, we use land-use regression (LUR) to identify the sources of contaminants and quantify their association with environmental responses (Messier et al., 2014). By constructing source terms that characterize contributions through spatial predictor models (SPMs), LUR leverages hyperparameters and databases of spatially distributed sources to describe the decayed range of influence around sources (Wiesner-Friedman et al., 2021b). LUR studies can increase the ecological understanding of how sources influence ARG levels and help develop microbial risk assessments for AMR (Nappier et al., 2020).

To the best of our knowledge, only two studies using LUR or SPMs have characterized source contributions to the AMR levels of rivers and their quantified associations (Pruden et al., 2012; Amos et al., 2015). Pruden et al. (2012) explored different SPMs that account for average upstream capacities and found that WWTPs and AFOs were associated with *sul1* relative abundance in sediment (2012). A recent study (Amos et al., 2015) modeled the decaying contributions [i.e., decaying concentrations of cellular and extracellular *intI1* (i.e., genes related to ARG mobility and pollution) and concentrations of selective pressures (e.g., antibiotics, biocides, microplastics, etc.)] coming from upstream WWTPs, leading to higher levels of *intI1* relative abundance in sediment.

These two LUR studies implemented different SPMs. Pruden et al. (2012) used the inverse distance-weighted (IDW) interpolation of pollution capacities upstream of the sampling location. This SPM is an interpolation of pollution capacities upstream of the sampling point. Therefore, it does not guarantee that pollution decreases away from sources, as would be expected from dilution and degradation processes. Amos et al. (2015) used a sum of exponentially decaying contributions (SEDC) applied to upstream sources. This model is different from the IDW interpolation in that it accounts for the density of sources, and it is such that the predictor value decreases away from sources, which is physically meaningful. Factors including dilution due to flow (Knapp et al., 2012), overland flow, and manure hauling from AFOs to application fields contribute to the dissemination of microbial contamination to rivers (Wiesner-Friedman et al., 2021a,b). Our goal is to expand upon previous modeling with a generalized SPM that incorporates these four components (i.e., density and proximity of upstream sources, overland flow, and dilution) to screen many potential sources of elevated AMR.

By implementing a LUR approach in a new region with a more generalized SPM, we aimed to (1) estimate relative abundance (i.e., ARG/16S rRNA gene) ratios (RARs) that express how AMR levels respond to the influence of different types of upstream sources, (2) characterize the overland range of influence around AMR sources, and (3) predict AMR levels at unsampled river sites. We were further interested in studying source impacts on riverbed sediment and surface water, representing time-integrated effects and transient contamination (Wiesner-Friedman et al., 2021a). To accomplish this, we applied the newly developed microbial *Find, Inform*, and *Test* (FIT) framework (Wiesner-Friedman et al., 2021b), which uses LUR and novel SPMs, and applied FIT to a panel of four ARGs (*ermB, tetW, qnrA*, and *sul1*) and one resistance-associated gene (*intI1*) (collectively called ARGs throughout) quantified from riverbed sediment and surface water from a mixed-use Great Lakes watershed area. The panel of ARGs in this study were selected for their associations with agricultural resistance [i.e., *tet(W)* and *erm(B)*], clinical significance (i.e., *qnrA*), and as mobile genetic elements (i.e., *intI1* and *sul1*) (Beattie et al., 2018). Three ARGs have been previously studied using LUR [i.e., *tet(W), sul1*, and *intI1*] but were only measured from sediment (Pruden et al., 2012; Amos et al., 2015), and two [i.e., *erm(B)* and *qnrA*] have not been studied with LUR. Compared to these previous studies, we modeled contributions from new types of spatially distributed sources (e.g., land-applied waste from municipal or industrial origins) and for ARG responses in sediment and surface water.

## 2. Methods

### 2.1. Sampling and sample analysis of antibiotic resistance genes

**Figure 1:**
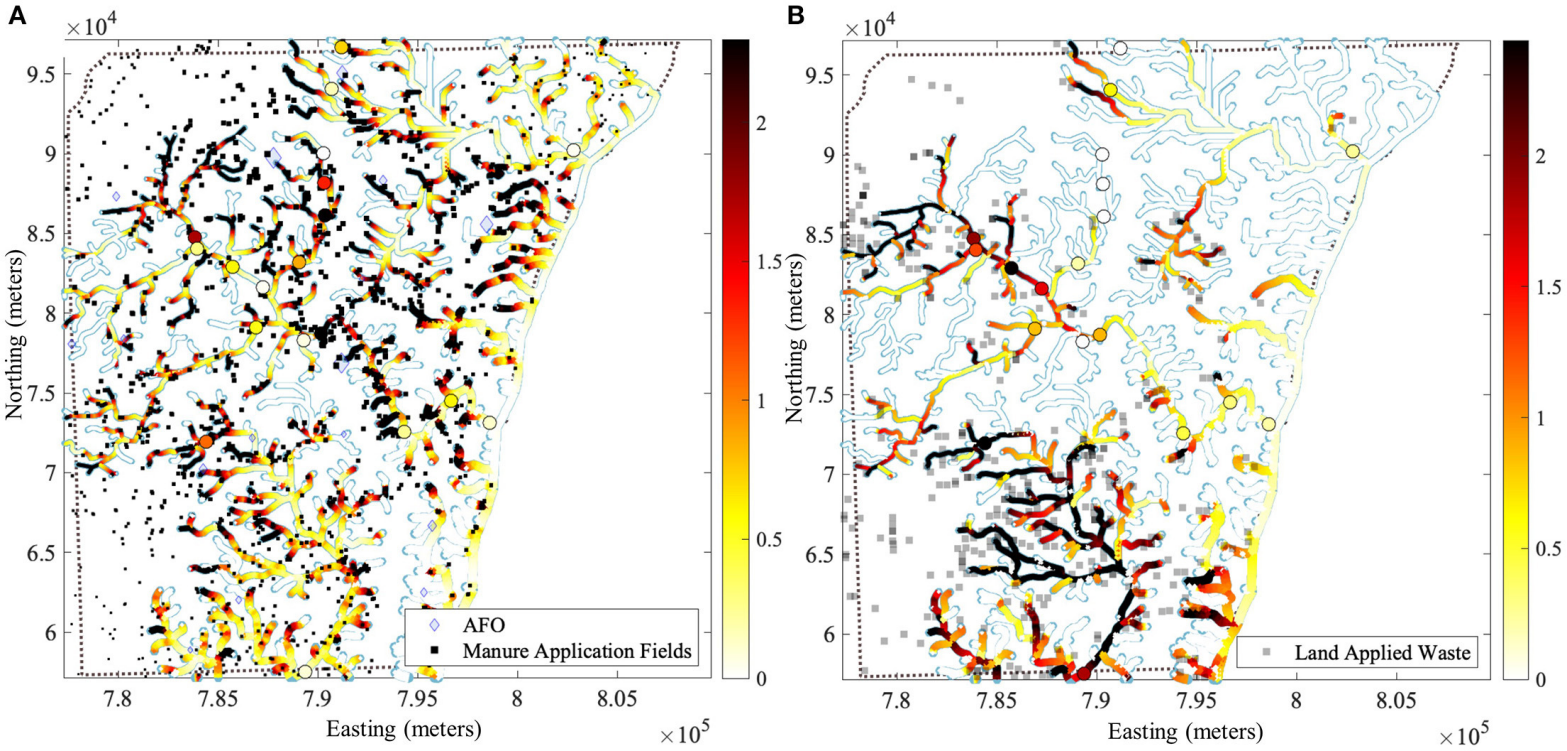
Conservative values of the standardized sum of exponentially decaying contribution (SEDC) are shown for two types of spatial prediction models: **(A)** AFOs *via* manure application at agricultural application fields, and **(B)** land application sites with municipal or industrial wastes and septage. These standardized SEDCs are conservative in using the maximum hyperparameter values α^(*u*)^ (see Equation 1) found across ARG responses. For subpanel a, we used the maximum hyperparameter values α^(*u*)^ obtained across three relative abundance responses [*erm(B), tet(W)*, and *sul1*] in sediment $\left[max(\alpha^{\left(u\right)})=\;\{\gamma_{G}=\;14\;km,\;\alpha_{O}=\;13\;km,\;\alpha_{R}=\;2\;km\}\right]$. For subpanel b, we used the maximum hyperparameter values α^(*u*)^ obtained across two relative abundance responses [*tet(W)* and *intI1*] in surface water $\left[max(\alpha^{\left(u\right)})=\;\{\alpha_{O}=\;10\;km,\;\alpha_{R}=\;10\;km\}\right]$. An increase of 1 on the color scale corresponds to a one standard deviation increase of the corresponding SEDC. The multiplication of the standardized SEDC shown here with a given regression coefficient β_*u*_ from Table 1 gives the increase in the corresponding log10 relative abundance *y*. Circles represent the sampling locations associated with this study and highlight the conservative value of the standardized SEDC at that site. For example, for *erm(B)* in sediment, the β_*u*_ for *u* = AFO *via* manure application fields is β_*u*_ = 0.199, therefore, a one standard deviation increase (shown in orange in the map) in the standardized SEDC for AFO *via* manure application fields increases *y* (the log10 relative abundance) by 0.199, or conversely, the relative abundance *z* increases by a relative abundance ratio (RAR) of $10^{\beta_{u}}=1.58$, which corresponds to a 58% increase in relative abundance *z*. Database representations are detailed in Supplementary material S7.

The data used for this study were originally collected and processed by Marquette University researchers (Beattie et al., 2018) based on (1) the number and spatial distribution of samples and (2) the high detection rate of ARGs in riverbed sediment and surface water. The samples were obtained from Kewaunee, Ahnapee, and East Twin Rivers in Kewaunee County, Wisconsin during five sampling events representing four seasons and 20 sampling sites (sampling sites are depicted as circles in Figure 1). Sites were selected based on the impacts of expected variability from the dense livestock agriculture in Kewaunee County and public access to the sites (Beattie et al., 2018). DNA was extracted from samples, and quantitative PCR was used to quantify AMR-associated genes [i.e., *erm(B), qnrA, tet(W), sul1*, and *intI1*] and the 16S rRNA gene. Antibiotic resistance gene selection is detailed in a previously published study (Beattie et al., 2018); briefly, genes resistant to antibiotics commonly used in agriculture (tetracycline, macrolides, and sulfonamides; *tet*(W), *erm*(B), and *sul1*), genes found on mobile genetic elements (*intI1* and *sul1*), and genes conferring resistance to clinically important antibiotics (fluoroquinolones; *qnrA*) were chosen to explore the diversity of environmental resistance in the study area. Values measuring below the detection limit (< 8% of the data points; see Supplementary Table S1) were set to half the detection limit. Detailed sampling methods, DNA extraction, qPCR protocols, and the full ARG dataset (relative and absolute abundances) can be found in a previously published study (Beattie et al., 2018). River network, precipitation, temperature, and source location data and processing can be found in previously published studies (Wiesner-Friedman et al., 2021a,b).

**Table 1:** Symbols and abbreviations from Equation 1.

**Term**	**Description**
*y*_*i*_	log10 of the relative abundance *z_*i*_*, where *z_*i*_* is the ratio of ARG copies to 16S rRNA gene copies for each *i^*th*^*sample.
*P*1_*i*_	Recent precipitation for each *i^*th*^*sample.
*P*2_*i*_	Antecedent precipitation for each *i^*th*^*sample.
*Freezing*_*i*_	Bernoulli distributed variable representing whether the monthly average temperature was freezing for each *i^*th*^*sample.
$s_{i}^{\left(u\right)}\left(\alpha^{\left(u\right)}\right)$	The impact on each *i^*th*^*sample from the *u^*th*^*source type modeled with the Sum of Exponentially Decaying Contributions (SEDC) Spatial Predictor Models (SPMs) that account for the ground hauling, overland, and river distance flow (Wiesner-Friedman et al., 2021a,b).
β_0_	The regression intercept.
β_1_	The increase in *y*_*i*_ for a one standard deviation increase in recent precipitation.
β_2_	The effect that a one standard deviation increase in antecedent precipitation has on the effect of recent precipitation β_1_.
β_3_	The effect of freezing temperatures on *y*_*i*_.
β_*u*_	The increase in *y*_*i*_ for a one standard deviation increase in the source term $s_{i}^{\left(u\right)}\left(\alpha^{\left(u\right)}\right)$.
ε_*i*_	The random error for each *i^*th*^*sample.

### 2.2. Physically meaningful land-use regression model

This study uses a physically meaningful LUR model (i.e., source terms are not allowed to have negative regression coefficients) implemented in FIT without modifiers (i.e., attenuators or amplifiers) to focus on characterizing source contributions to each ARG response, *y*_*i*_ (Wiesner-Friedman et al., 2021b):


(1) $$\begin{array}{l} {\text{y}}_{i}\;=\;\beta_{0}+P1_{i}\left(\beta_{1}+\;\beta_{2}P2_{i}\right)+\;\beta_{3}Freezing_{i}\\ \;+\left\{\sum_{u=1}^{U}\beta_{u}s_{i}^{\left(u\right)}\left(\alpha^{\left(u\right)}\right)\right\}+\varepsilon_{i} \end{array}$$

The ARG response, *y*_*i*_, represents log10 of the relative abundance *z*_*i*_, where *z*_*i*_ is the ratio of ARG copies to 16S rRNA gene copies. The first three variables, namely, *P*1_*i*_, *P*2_*i*_, and *Freezing*_*i*_, represent recent precipitation, antecedent precipitation, and a seasonal, Bernoulli-distributed variable representing whether the monthly average temperature was freezing for each *i*^*th*^sample. The first term, β_0_, and the last term, ε_*i*_, represent the regression intercept and random error, respectively. β_1_, β_2_, and β_3_ are associated regression coefficients, and $\overset{U}{\underset{u=1}{\sum }}\beta_{u}s_{i}^{\left(u\right)}\left(\alpha^{\left(u\right)}\right)\;$is a sum of contributions from spatially distributed sources. Each source term, $s_{i}^{\left(u\right)}\left(\alpha^{\left(u\right)}\right),\;$is defined as a function of ground hauling, overland, and downstream decay hyperparameters; α^(*u*)^, the flow-connected distances (i.e., overland and downstream distances) from the spatial locations of sources of type *u* (e.g., WWTPs) to the sampling locations of each *i*^*th*^sample; and sample site flow (proxied by Strahler stream order) (Wiesner-Friedman et al., 2021b). The sources can also be weighted by information associated with their scale (e.g., size of the land cover area, gallons of manure, or equally weighted) (Wiesner-Friedman et al., 2021a). Because hyperparameters describe the distance decay of dimensionless quantities that denote the scale of sources, source terms describe dimensionless source contributions to sampling sites. All source terms $s_{i}^{\left(u\right)}$ are SPMs equal to the z-scored Sum of Exponentially Decaying Contributions (SEDC) so that a one standard deviation increase in the *u*^*th*^ SEDC represents a β_*u*_ increase in the response. An RAR expresses the ratio of relative abundances for a one standard deviation increase in source *u*. In other words, a one-unit increase in SPM $s_{i}^{\left(u\right)}$ results in a (100(*RAR*^(*u*)^−1)) percentage increase in relative abundance *z* (Wiesner-Friedman et al., 2021b). See Supplementary material S5 for details.

### 2.3. Data for spatially distributed sources

Multiple spatial databases are available to represent potential sources in Kewaunee County. While many sources of elevated ARGs exist globally, we identified thirteen categories of potential sources that may be important to Kewaunee County (*u* = 1,2,…,13).

The first eight potential sources of the *u*^*th*^ categories were spatially related to sampling site locations with SPMs, which are the z-scored overland and river-distance with flow (ORF) SPM: (1) AFOs, (2) manure application fields, (3) septic systems, (4) industrial land application sites, (5) municipal or septage (i.e., domestic-originating) land application sites, (6) WWTPs, (7) low-intensity developed land cover representing rural accumulations on imperviousness, and (8) high-intensity developed land cover representing more urban/residential accumulations on imperviousness. The next three potential sources were soil sources represented by the dominant soil type within a 1-km radius of the sampling location: (9) Type A represents soils with the highest infiltration rate when saturated, likely consisting of sand, sandy loam, loamy sand, or gravel soil types; (10) Type C represents soils with a low infiltration rate when saturated, likely consisting of clay loam, silty clay loam, sandy clay, or silty clay; and (11) Type D represents soils with a very low infiltration or high run-off potential, likely consisting of clay loam, silty clay loam, sandy clay, or silty clay. The last two source categories were those related to the sampling site locations using the ground hauling, overland, and river distance with flow (GORF) SPM. Here, the SPM leverages two source locations to capture how land application occurs on land disproportionately closer to the origin of the waste [e.g., manure is hauled from AFOs (*u* = 1) to manure application fields (*u* = 2), and AFOs will minimize hauling distances for cost purposes (Hadrich et al., 2010)]. The sources defined with this SPM are 12) AFOs *via* the ground transport or hauling of manure to fields and 13) domestic-originating land application sites (*u* = 5) with waste that is applied more greatly in proximity to denser residential areas represented by septic system locations (*u* = 3). See previous study for SPM equations and descriptions (Wiesner-Friedman et al., 2021b).

### 2.4. Application of the FIT framework to ARG responses

The microbial FIT framework is a three-step approach (Wiesner-Friedman et al., 2021b) using the physically meaningful LUR (Equation 1). The three stages of the FIT framework, *Find, Inform*, and *Test*, were applied independently to the 10 ARG responses (i.e., sediment and water measurements of five ARGs). This modeling was implemented in MATLAB 2020b (https://scicrunch.org/resolver/RRID:SCR_001622).

In *Find* reliable databases of spatially distributed sources, many candidate databases can be explored for their ability to reproduce source-term relationships for unseen data. Here, for each of the potential ARG source categories (*u* = 1,2,…,13), we explored the candidate options for representing the sources based on (a) available databases [e.g., the Wiscland-2 land cover database, the Wisconsin Pollution Discharge Elimination System (WPDES) database, and county databases available for manure storage and septic systems], (b) coding options, (c) different classes of data, and (d) weighting options (see Supplementary material S2 for details).

The *find* stage obtains a reliability score for each candidate database option. The reliability score is calculated by obtaining hyperparameters for the SPM for a training set of response data. Using training hyperparameters for a test set (see Supplementary material S4), we calculated a reliability score with test-set regression coefficients. The score rewards candidate databases with the most consistently positive test-set regression coefficients [i.e., sign stability score (SSS)], the largest sum of test-set coefficients (*M*), and the lowest variability of the test-set coefficients (Wiesner-Friedman et al., 2021b). See Supplementary material S2 for the reliability score equation.

In the *inform* stage, source terms are informed with transport-characterizing hyperparameters in ORF and GORF SPMs. In this stage, hyperparameter values, **α**^**(*u*)**^ (i.e., describing average ground hauling distance γ_*G*_, decay overland α_*O*_, and downstream α_*R*_), were obtained by independently maximizing the RAR (10^β^(*u*)) for every source type. This maximization was subject to a penalty on very low or high values of overland decay and ground hauling hyperparameters, α_*O*_ and γ_*G*_, that yielded poor regression qualities (Wiesner-Friedman et al., 2021b). The spatial predictor models leveraging the hyperparameters, **α**^**(*u*)**^, are described in detail in the microbial FIT framework (Wiesner-Friedman et al., 2021b).

The goal of the last stage is to *test* the predictive ability of informed source terms to identify key sources of elevated ARGs in Kewaunee County Rivers and Streams. Before model selection, if the correlation of informed source terms was large (ρ≥0.7), those yielding the highest univariate R-squared were chosen over correlated options (Dormann et al., 2013). Then, seasonal, precipitation, and source terms were stepwise selected with AIC. The prediction of individual source impacts on unsampled locations was initially assessed through the *find* stage based on the robustness of the source term's consistent contributions and the ARG's log10 relative abundance across training and test sets. Ultimately, the prediction of total source impacts was assessed with the adjusted R^2^ resulting from the *test* stage.

### 2.5. Conducting interviews/surveys with Wisconsin dairy cattle veterinarians

To understand which ARGs are likely to be shed by bovines based on antibiotic usage, we reviewed information from a thorough systematic review outlining and quantifying antibiotic usage on Wisconsin dairy farms (Pol and Ruegg, 2007). Since the publishing of that study, practices may have changed, and a previous ARG study found local knowledge to be beneficial (Rogers et al., 2018). We conducted a small survey with dairy cattle veterinarians and asked Wisconsin dairy cattle veterinarians about the changes they have observed in antibiotic use over their careers. Google Maps was used to search for veterinarians, veterinary services, and “large animal” services. In total, 12 veterinary offices were identified as serving the dairy cattle industry, representing the practices of over 30 veterinarians. Surveys were sent to all 12 offices, and four returned our surveys. See Supplementary material S6.1 for survey questions and veterinarian responses.

## 3. Results

### 3.1. The ARGs in this study are biologically linked to antibiotic usage in Wisconsin AFOs

According to the four veterinarians we interviewed, ceftiofur, a beta-lactam (Dowling, 2004), was the most frequently prescribed antibiotic for disease treatment (Dowling, 2004). Other antibiotics that veterinarians prescribe are enrofloxacin, florfenicol, tulathromycin, and oxytetracycline. These interviews (see Supplementary material S4 for interview details and discussion) reflected antibiotic use for disease treatment that was consistent with the findings of the 2007 study on antibiotic usage at AFOs in Wisconsin (Pol and Ruegg, 2007). These antibiotics belong to the broader classes of fluoroquinolones, sulfonamides, macrolides, and tetracyclines, which correspond well with resistance encoded by or co-occurring with the ARGs used for this LUR modeling (Pal et al., 2015; Beattie et al., 2018). Our interviews did indicate that some antibiotics are still used preventatively (e.g., tetracycline flushes) because operations have difficulty monitoring large herds. Preventative antibiotic use is therefore a missing component in understanding the frequency and dose associated with these classes of antibiotics. However, our interviews and the 2007 study provide a biological link between the panel of ARGs from our study and dairy AFOs. Additionally, although *intI1* has been identified as a marker for anthropogenic pollution more broadly, clinical class I integrons can carry ARG cassettes, conferring multidrug resistance with the ability to spread rapidly through horizontal gene transfer (Gillings et al., 2015).

We could not directly obtain information on clinical antibiotic usage in rural Wisconsin. However, one wastewater study (Karthikeyan and Meyer, 2006) indicates a total of six classes of antibiotic compounds found in the influent of municipal wastewater from across Wisconsin. Based on the frequency of the detection of different antibiotic classes, the study indicates that the ARGs for this study may also well represent the anthropogenic impacts of clinical antibiotic use.

### 3.2. Overland and downstream flow from bovine sources consistently contribute to elevated ARGs

After implementing the FIT framework across five ARGs in sediment and surface water, we found that all five of the ARG responses (see Table 2) are positively associated with bovine sources (i.e., AFOs, AFOs *via* the ground hauling of manure to application fields, or manure application field locations) in at least surface water or sediment. For ARG responses in *sediment*, FIT selected the GORF AFO source term, which represents the contributions from AFOs *via* the ground hauling of manure to application fields. A one standard deviation increase in GORF AFO contributions was associated with RARs of 1.58 and 2.01, representing 58% and 101% increases (*p* < 0.10) in the relative abundance of *erm(B)* and *tet(W)*.

**Table 2:** Regression results for predicting the relative abundance of *erm(B), tet(W), qnrA, sul1, and inI1* (log10 gene copies per 16S-rRNA copies) in riverbed sediment (columns toward the left) and surface water (right-most five columns).

**Environmental matrix**		**Riverbed sediment**				**Surface water**				
**ARG (*****n** =* **sample size)**		***erm(B)*** **(*****n** =* **91)**	***tet(W)*** **(*****n** =* **91)**	***sul1*** **(*****n** =* **91)**	***intI1*** **(*****n** =* **91)**	***erm(B)*** **(*****n** =* **98)**	***tet(W)*** **(*****n** =* **98)**	***qnrA*** **(*****n** =* **98)**	***sul1*** **(*****n** =* **98)**	***intI1*** **(*****n** =* **98)**
**Recent precip**.	Std. Regression Coefficient (**β**_**1**_)	−0.723^**^	0.654^**^	NS	−0.330^**^	0.415^**^	0.635^**^	0.213^**^	0.637^**^	0.277^**^
**Recent x antecedent precip**.	Std. Regression Coefficient (**β**_**2**_)	0.561^**^	0.923^**^	NS	0.256^**^	−0.394^**^	−0.300^**^	−1.10^**^	NS	0.637^**^
**Freezing**	Regression Coefficient (**β**_**3**_)	−0.621^*^	NS	NS	−0.801^**^	1.94^**^	NS	NS	NS	NS
**Bovine sources**	Bovine Source Description	GORF AFO (*via* ground hauling of manure to application fields)	GORF AFO (*via* ground hauling of manure to application fields)	NS	NS	GORF AFO (*via* ground hauling of manure to application fields)	ORF AFO	ORF AFO	ORF Manure app. fields	ORF Manure app. fields
	Std. Regression Coefficient (**β**_**u**_)	0.199^*^	0.303^*^	NS	NS	0.162^**^	0.247^**^	0.173^**^	0.148^*^	0.134
	RAR ($10^{\beta_{u}}$)	1.58^*^	2.01^*^	NS	NS	1.45^**^	1.77^**^	1.49^**^	1.41^*^	1.36
	Influence Range (**α**_**O**_)	< 13 km				< 10 km				
**Land-app. waste sources**	Land applied waste Source Description	NS	NS	Land-applied waste- *residential*	Septage ground transport to land app. sludge- *residential*	NS	Land-applied waste- *industrial*	NS	NS	Land-applied waste- *industrial*
	Std. Regression Coefficient (**β**_**u**_)	NS	NS	0.211^**^	0.155^*^	NS	0.134^*^	NS	NS	0.148^*^
	RAR ($10^{\beta_{u}}$)	NS	NS	1.63^**^	1.43^*^	NS	1.36^*^	NS	NS	1.41^*^
	Influence Range (**α**_**O**_)	< 8 km				< 10 km				
**Soil sources**	Soil Source Description	NS	NS	Type A (Sand, Sandy loam, Loamy sand, and Gravel)	Type A (Sand, Sandy loam, Loamy sand, and Gravel)	Type D (Clay loam, silty clay loam, sandy clay, and silty clay)	NS	NS	NS	NS
	Regression Coefficient (**β**_**u**_)	NS	NS	0.402^**^	0.253	0.231	NS	NS	NS	NS
	RAR ($10^{\beta_{u}}$)	NS	NS	2.52^**^	1.79	1.70	NS	NS	NS	NS
	Influence Range (**α**_**Radius**_)	1 km								

^*^p < 0.10, ^**^p < 0.05. NS indicates that no terms were selected for the source category. Other source categories not selected: wastewater treatment plants (WWTPs), septic systems, and developed land cover. No terms were selected for the log10 relative abundance of qnrA in sediment. The sample size is indicated for each of the responses in each column. For each of the climatic and source terms, the standardized regression coefficient, **β**, is provided resulting from the Test stage of FIT. For each source term, two additional rows result from the Find and Inform stages of the FIT framework. For each source term category (i.e., bovine, land-applied waste, or soil), the source description, the relative abundance ratio (*RAR* = 10^β^), and hyperparameters indicating the influence range around sources, **α**, are summarized. Precipitation-term associations are shown in blue. The associations with freezing temperatures are shown in white. Bovine source associations are in red. Land-applied waste sources are shown in yellow.

Across ARG responses in *surface water*, FIT selected the GORF AFO source term for *erm(B)*. A one standard deviation increase in these AFO contributions was associated with a 45% (*p* < 0.05) increase in the relative abundance of *erm(B)* in surface water. For *tet(W)* and *qnrA* responses, FIT selected the ORF AFO source term, representing the contributions directly from AFO locations. A one standard deviation increase in AFO contributions was associated with 77% (*p* < 0.05) and 49% (*p* < 0.05) increases in the relative abundance of *tet(W)* and *qnrA*, respectively. For *sul1* and *intI1*, FIT selected the ORF manure application fields representing contributions directly from field locations (i.e., irrespective of AFOs). A one standard deviation increase in manure application field contributions was associated with 41% (*p* < 0.10) and 36% (inclusion lowers AIC) increases in the relative abundance of *sul1* and *intI1*, respectively.

In sediment and surface water, the magnitude of the association between *tet(W)* and AFOs was greatest compared to other ARG responses. A greater association may indicate that more *tet(W)* genes are located at AFOs or may suggest that oxytetracycline is used frequently to prevent diseases at dairy AFOs compared to other antibiotics. Some studies suggest that *tet(W)* and tetracycline resistance may be more specific to dairy feces than other ARGs and antibiotic resistance phenotypes (Srinivasan et al., 2008; Kyselková et al., 2015). The strength of the associations from our study supports that *tet(W)* is correlated with dairy manure.

This is the first study to characterize ARG contributions with GORF or ORF SPMs for bovine sources. Our findings imply that overland and downstream transport and dilution from flow are key processes in disseminating AMR from AFOs and manure fields. This study also suggests that manure hauling to application sites is a factor in elevated ARGs in the environment. Our novel spatial predictors and modeling approach are in agreement with AFO's association with ARGs found by Pruden et al. (2012) using a different SPM and applied to a different geographical region. Pruden et al. (2002) found that the relative abundance of *sul1* in *sediment* was positively correlated (R^2^ = 0.35, *p* < 0.001) with the average upstream capacities of AFOs. However, these authors found no significant relationship with the relative abundance of *tet(W)* in sediment (Pruden et al., 2012), which could reflect differences in livestock and antibiotic use. Our study quantified seven novel associations between bovine sources and ARG responses. We have quantified the associations between bovine sources and levels of *erm(B), tet(W), qnrA, sul1*, and *intI1* in surface water (i.e., five novel associations) and the association between bovine sources and levels of *erm(B)* and *tet(W)* in sediment (i.e., two novel associations), which can inform ecological studies of AMR and microbial risk assessment.

### 3.3. Flow affects consistent detection of signals from bovine sources

The *find* stage of FIT enables the exploration of many databases of collocated, spatially distributed sources. The result is the set of source locations and associated information that consistently represent sources to the response from a cross-validation approach. Here, we report the *find* stage results for bovine sources because they were the most consistently selected source from the *test* stage of FIT. Across ARG responses in surface water, FIT modeled contributions from AFOs with the county manure storage option and contributions from manure application fields with the crop rotation land cover option.

For ARG responses in *sediment*, FIT modeled contributions from AFOs with WPDES CAFO locations, but differences existed in CAFO weightings. The difference in weighting CAFOs by animal units for *erm(B)* and *tet(W)* and equal weighting for *sul1* may reflect differences in the transport processes resulting in elevated relative abundance, independent of the number of animal units (e.g., some other selective pressure emanating from sources).

One key difference in FIT's database selection was that, to represent AFOs, the WPDES CAFOs option was selected for sediment responses and the county's manure storage option for surface water responses. One explanation for this difference is that sediment sampling was impossible at three sites during one high-flow event (Beattie et al., 2018). These manure storages are known to overflow during high-flow events (Burch et al., 2021), and the additional surface water samples may have better captured transient contamination from manure storages.

### 3.4. Land application of septage, municipal, and industrial waste is another source of elevated ARGs in the environment

After implementing FIT across 5 ARGs in sediment and surface water, we found that three of the ARG responses (Table 2) are positively associated with land-applied waste sources in sediment (*sul1* and *intI1*) or surface water [*tet(W)* and *intI1*]. ARG responses in *sediment, municipal waste*, or *septage* land application characterized land-applied residential waste sources. For *sul1*, FIT selected the ORF land-applied residential waste source term representing land-applied waste from residential origins (i.e., land application of municipal waste or septage consisting of solid or semi-solid residue generated during the treatment of domestic sewage *via* primary, secondary, or advanced wastewater treatment and the wastewater contents of septic or holding tanks, dosing chambers, grease interceptors, seepage beds/pits/trenches, privies, or portable restrooms (Wis. Admin, 2021). A one standard deviation increase in contributions from land-applied waste from residential use was associated with a 63% (*p* < 0.05) increase in the relative abundance of *sul1*. For *intI1*, FIT selected the GORF land-applied waste source term, representing land-applied residential waste weighted by the density of nearby septic systems. A one standard deviation increase in land-applied waste from residential use was associated with a 43% (*p* < 0.05) increase in the relative abundance of *intI1*.

For ARG responses in *surface water*, industrial waste's land application characterized these five ARGs' secondary sources. For *tet(W)*, FIT selected the ORF land-applied industrial waste source term representing by-product solids from the animal product or food processing industry (i.e., remains of butchered animals, paunch manure, cheese production waste, and vegetable waste materials). A one standard deviation increase in land-applied waste from industrial use was associated with a 36% (*p* < 0.10) increase in the relative abundance of *tet(W)*. Then, for *intI1*, FIT selected the ORF land-applied industrial waste source term [i.e., “both the by-product solids from the animal product or food processing and liquid waste such as silage, leachate, whey, whey permeate, whey filtrate, contact cooling water, cooling or boiler water containing water treatment additives, and wash water generated in industrial, commercial, and agricultural operations” (Wis. Admin, 2021)]. A one standard deviation increase in land-applied waste from industrial use was associated with a 41% (*p* < 0.10) increase in the relative abundance of *intI1*.

Our findings are consistent with current knowledge that ARGs are enriched in biosolids from treatment processes (i.e., primary, secondary, or advanced wastewater treatment) (Chen and Zhang, 2013; Burch et al., 2014; Pepper et al., 2018). This is the first study to report an association between modeled contributions from spatially distributed land-applied waste and ARGs recovered from riverbed sediment and surface water. This is also the first study to show that septage and municipal or industrial waste disposal on land pollute and correspond with a quantifiable environmental impact on antibiotic resistance levels in sediment and surface water. The WPDES database lists the facility names associated with the land application sites. After searching on company websites for the products associated with each facility producing industrial wastewater or sludge destined for land application, we found that 88.8% of the industrial land-applied waste sites are associated with dairy and meat products. None (i.e., 0%) of the facilities were associated with pharmaceuticals. This suggests that the disposal of industrial wastes from dairy and meat processing extends the polluting ability of industrial livestock agriculture and that industrially produced, land-applied pharmaceutical waste is not a source of elevated ARGs in this region.

### 3.5. Sandy soils are associated with sediment ARGs, and clay soils are associated with surface water ARGs

The bottom of Table 2 shows the results from the Inform and Test stages corresponding to the soil as a source. For surface water responses, only soil type D (clay loam, silty clay loam, sandy clay, or silty clay) coverage was associated with a 70% increase in the log10 relative abundance of *erm(B)*. In sediment responses, soil type A (i.e., sand, sandy loam, loamy sand, or gravel) coverage was associated with 152% and 79% increases in *sul1* and *intI1* log10 relative abundances, respectively.

Soil type D contains clay, and we expect ARGs to correlate with clay based on previous research (Mao et al., 2014; Wang et al., 2016). A previous study also suggests that clay microbial communities are more resilient to change from anthropogenic sources compared to other soil types, like sand (Neumann et al., 2013). Therefore, this contribution to *erm(B)* relative abundance of clay soil sources may suggest the long-term impacts of agricultural sources on clay microbial communities. However, we found different results from the surface water results. Overall, soil appears to significantly contribute to the relative abundance of these ARGs, and the differences in soil sources for surface water and sediment in this study have many potential explanations (e.g., adsorption, desorption, and absorption between overland soil, riverbed sediment, and surface water). More observational data and controlled mesocosm-scale experiments are needed to validate these findings and characterize these complex dynamics.

### 3.6. Regional differences may affect the primary sources of elevated ARGs

WWTPs were expected to show associations with *sul1* and *intI1*, as *sul1* is often conserved in integron-integrase mobile genetic elements (Pruden et al., 2012; Amos et al., 2015), but the FIT model selected neither municipal nor industrial WWTPs associating with any of the five relative abundance responses across surface water or sediment. One explanation is that in this rural area of ~20,000 people (US Census Bureau, 2022), only five of the WWTPs were flow-connected to the 20 sampling sites. Our study area consists of a larger bovine–human ratio (Borchardt et al., 2021; Burch et al., 2021; Wiesner-Friedman et al., 2021a) compared to the study areas of previous ARG research in the South Platte River Basin in Colorado, United States (Pruden et al., 2012), and the Thames Watershed in Oxfordshire, United Kingdom (Amos et al., 2015).

In our study, both *sul1* and *intI1* were associated with land-applied municipal waste and septage. The land-applied municipal waste and septage represent the aggregation of treated septage and wastewater, suggesting that some ARGs may originate from WWTPs, but most likely, the persistent application of biosolids on land represents a more significant source than WWTP effluent or septic systems in this region. Quantitation methods with low detection limits for *intI1*, more flow-connected sampling sites to sources, or a different ARG panel may be needed to detect the impacts of WWTP effluent or septic systems.

### 3.7. The overland influence range around sources extends up to 13 km

Bovine source terms associated with elevated ARGs in *sediment* had exponential influence ranges of α_*O*_**<**13 km on the river network, indicating that decayed contributions would still be detected in the river when manure fields were up to 13 km away from the river network. For all bovine source terms associated with ARG responses in *surface water*, decayed contributions would still be detected when sources were up to 10 km from the river network. For all land-applied waste source terms associated with ARGs, the influence range was up to 8 km for *sediment* and 10 km for *surface water* responses.

These overland, exponential influence ranges, α_*O*_, were determined from the *inform* stage of FIT. In a previous study (Wiesner-Friedman et al., 2021a), we remarked that this hyperparameter captures more than average overland transport. This hyperparameter characterizes the extent to which a microbial response can capture a signal from sources so that longer overland influence ranges may indicate either A) longer transport and B) an increased probability of detection, or both. Previously, we found longer overland influence ranges around sources for host-associated *Bacteroides* in sediment (i.e., α_*O*_> 1 *km*) vs. surface water (i.e., α_*O*_ < 1 *km*) (Wiesner-Friedman et al., 2021a).

In this study, on average, we found long overland influence ranges [i.e., a mean value of $\hat{\alpha_{O}}=$7.07 km (95%CI: 4.22 km, 9.92 km)] for both sediment and surface water. ARGs can exist naturally in soils (D'Costa et al., 2011) and be carried by either aerobic or anaerobic bacteria (Xu et al., 2021); They can be transferred to other bacteria by several genetic mechanisms (Davies and Davies, 2010). These factors may increase the transport of and ability to detect ARGs in surface waters compared to anaerobic *Bacteroides* gene markers. Furthermore, *Bacteroides* persist briefly outside of the gut of their hosts (Ballesté and Blanch, 2010), so shorter overland influence ranges would be expected. One implication of longer influence ranges around bovine sources for ARGs compared to host-associated *Bacteroides* genes is that host-associated markers may underrepresent the risks associated with fecal contamination from bovine sources in surface water. Additionally, this research and previous studies have found an increased probability of detecting microbial genes in sediments compared to surface water (see Supplementary material S2) (Kasich et al., 2012; Wiesner-Friedman et al., 2021a), which suggests that risks associated with particular sources may be underrepresented from sampling transient surface water compared to time-integrated polluted sediment.

The FIT model of ARGs in the environment is the first to characterize overland influence ranges around these sources. A setback distance of 34–67 m from surface water has previously been recommended for manure and slurry land application under experimental conditions (Hall et al., 2020). The exponential influence ranges from our study indicate that sources very close to the river will greatly contribute but that distant sources up to 13 km away from the river network can also impact ARG levels. A factor that may lead to long influence ranges in this region is the karst geology, where fractures, sinkholes, caves, disappearing streams, and springs may provide direct pathways for contaminants, including antibiotics and ARGs, to reach ground and surface waters (Stange and Tiehm, 2020; Xiang et al., 2020). Our findings are consistent with a study in a karst region in Germany, where elevated ARGs and human-specific fecal markers were detected in a spring 9 km away from the suspected source (Stange and Tiehm, 2020).

### 3.8. The modeling predicts localized impacts to elevated ARGs in sediment and dispersed impacts in surface water

Since this is the first study to report LUR results for *erm(B)* in sediment and *erm(B), tet(W), qnrA, sul1*, and *intI1* in surface water, we identified the databases that most reliably report the spatial location of bovine and land-applied waste sources associated with elevated relative abundance of these ARGs and their corresponding regression coefficients (Table 2), and we have shown the spatial impact of these pollution sources (Figure 1).

Figure 1A shows the geographical distribution of an increase expected in the relative abundances of *erm(B), tet(W)*, and *sul1* in sediment associated with risk-conservative contributions from AFOs *via* the application of manure. This figure, which results from a GORF SPM, suggests that elevated ARGs in riverbed sediment are localized around manure application fields. However, the extent to which those sources (*viz*., differently sized black squares) qualify as polluters relates to their proximity to AFOs and the scale of the operation (viz. differently sized blue diamonds). This localized pollution may be influenced by ARG soil attachment (Barrios et al., 2021). In previous LUR, AFOs were associated with *sul1* in sediment, but the spatial localization was not reported or depicted (Pruden et al., 2012). Our LUR/FIT modeling showing localized sediment ARG pollution is consistent with the field studies of the enrichment of ARGs in AFO manure application and dissemination into the environment (Fahrenfeld et al., 2014; Wallace et al., 2018).

Figure 1B shows the geographical distribution of an increase expected in the relative abundances of *tet(W)* and *intI1* in surface water associated with risk-conservative contributions from land application sites. This figure, which results from an ORF SPM, suggests that land application sites have a dispersed impact on elevated ARGs in surface water.

These maps indicate locations where additional monitoring may be needed to understand the impacts of different sources on environmental and public (i.e., water users') health. The upper-bound hyperparameter values and database information also serve other regions with similar geography, land use, agricultural practices, and population to preliminarily define monitoring locations for AMR studies.

## 4. Discussion

This is the first LUR study of ARGs in surface water and the first LUR of more than two ARG responses from sediment samples. Our primary finding is that bovine sources (i.e., AFOs and manure application fields) were consistent sources of elevated ARGs. This continues the large body of work that has detected ARGs in livestock manure and slurry, on soil where the manure or slurry is applied, and downstream of livestock operations (Joy et al., 2013; Fahrenfeld et al., 2014; Peng et al., 2017; Wepking et al., 2017; Guo et al., 2018; Lopatto et al., 2019; Hall et al., 2020; Miller et al., 2020), but it is the first to connect modeled transport from manure land application to elevated AMR.

Previous research has revealed that domestic and industrial wasteland application sites are potential sources of elevated ARGs (Bondarczuk et al., 2016; Murray et al., 2019). However, our study distinguishes itself as one that found associations with the collective contributions from spatially distributed land-applied waste sites over a large spatial scale and measured ARG levels in the environment. A large body of work has detected microbial contaminants associated with land-applied wastes in soils, groundwater, surface water, and the air near application sites (Brooks et al., 2007; Lapen et al., 2008; Tanner et al., 2008; Edwards et al., 2009; Gottschall et al., 2009; Zerzghi et al., 2010; Esseili et al., 2012; Mohapatra et al., 2016; Pepper et al., 2019). Our research adds to this body of evidence.

While the US Environmental Protection Agency's (EPA's) Part 503 rule (Walker et al., 1994) regulates treatment and land application standards for class A and B biosolids, there are many similarities between pre-treatment class B biosolids and anaerobically treated manure or slurry from dairy CAFOs. Antibiotic-resistant bacteria, endotoxins, prions, pathogenic bacteria, and protozoa have been reported in both (Pepper et al., 2019). Few regulations exist for the treatment of livestock facility waste and its application on land. Regulations of livestock waste have focused on nutrient management, do not require treatment for viruses and pathogens, and only apply to be permitted CAFOs. Class B biosolids must meet minimal treatment and land application requirements. Therefore, one explanation for the increased consistency of association between bovine sources and ARG responses compared to land-applied waste sources is that this land application is more regulated compared to manure and that this additional regulation of treatment and land application locations has resulted in less harm to water quality than bovine manure land application. Another explanation could be that the quantity of bovine manure land application is much greater than other types in this region. However, regulators may want to evaluate current treatment standards, land application restrictions, and environmental monitoring of land application of waste more generally to improve water quality.

The dissemination of fluoroquinolone-associated resistance encoded by both chromosomal and plasmid-derived *qnrA* may increase the risks of quinolone-resistant human pathogens in surface water from plasmid-mediated HGT (Cummings et al., 2011). In the United States, fluoroquinolones are among the most common clinically prescribed classes of antibiotics (Antibiotic Use in the United States, 2017). Our findings that connect AFOs to the levels of *qnrA* in surface water are concerning, and the United States may want to consider broader enforcement of the recent policy limiting the use of antibiotics that are clinically relevant to humans in livestock settings (FDA, 2022).

Aside from the anthropogenic sources, the soil source results in this study support that the processes involving the interaction of anthropogenic sources, nearby soils, sediment, and surface water are too complex for a linear modeling approach to describe. The importance of soil sources in this study shows that characterizing natural processes (i.e., physical, biological, ecological, physicochemical, and chemical dynamics) may help predict ARG levels and mitigate the impacts of anthropogenic sources on AMR. Monitoring the impacts of chronic and acute ARG-associated pollution events (i.e., pollution with antibiotic-resistant bacteria and selective pressures) on adsorption, desorption, and absorption processes for various soil types may provide an additional benefit. More complex modeling calls for refinement in spatial scale and improvement in the prediction of soil characteristics.

A concern for the applicability of this study to other regions may be the availability of spatial databases. However, we found that a strength of this study is that the key ARG sources in this study (i.e., CAFOs, class A and B land application sites, and soil type) are represented by nationally available databases in the United States. Expanding these databases to include industrial livestock land applications and greater detail about application methods and the types of applied waste may be beneficial.

Here, we have focused on spatial relationships. However, we have modeled the impact of freezing temperature and antecedent precipitation (see Table 2 for results and Supplementary material S5 for details). We found that freezing temperature is negatively associated with ARGs in sediment and positively associated with one ARG in surface water, which is consistent with a previous study that found higher ARG abundances during the Wisconsin manure application season (Beattie et al., 2018). In addition, we identified three patterns of association for ARGs with antecedent precipitation (see Supplementary material S5). However, due to the temporal resolution of sampling approximately only once every 3 months, our results can only be interpreted as seasonal effects rather than impacts from recent and antecedent precipitation.

Additionally, due to this temporal resolution, our models may only capture the long-tail decay of ARGs disseminating from sources (Burch et al., 2014; Lopatto et al., 2019; Macedo et al., 2020; Barrios et al., 2021), meaning that peak contamination corresponding to periods directly following manure application is not well-characterized. Sampling at a finer temporal resolution could help to better capture these peaks as well as the impact of different flow events and meteorological variables.

The reproducibility of associations between the three source categories associated with increases in ARGs (i.e., bovine, land-applied waste, and soil) and the ARG responses in sediment and surface water provides evidence that elevated ARGs in the environment are linked to natural occurrence soils and land-applied wastes of bovine, residential, or industrial origins. In our study, we found that a one standard deviation increase in source impacts is associated with increases between 36 and 152%. This is larger than the expected percentage increase (17%) in total relative abundances of ARGs (TARG) in sediment and surface water associated with a total antibiotic selection pressure (TASP) score of 1–2 reported from a meta-analysis (Duarte et al., 2019). The greater association in our study may indicate the combined influence of enrichment from organic matter, antibiotics, and intracellular or extracellular ARGs disseminating from sources (Xie et al., 2018). Our findings call for more robust treatment regulations to remove or reduce ARBs and ARGs from wastes and policies to decrease antimicrobial use in livestock and humans.

Due to measured negative changes to the environment and public health of communities living nearby dense industrial livestock agriculture and land application sites (Greger and Koneswaran, 2010; Lowman et al., 2013; Hooiveld et al., 2016), a collaborative One Health approach (Robinson et al., 2016) may be beneficial for evaluating the impacts of these sources of mixed contaminants (i.e., pathogens, ARB and ARGs, heavy metals, disinfectants, fire retardants, pharmaceuticals, and polycyclic aromatic hydrocarbons) (Kinney et al., 2006; Ma et al., 2011; Pepper et al., 2018; Murray et al., 2019) on the shared health of humans, animals, and the environment.

## Data availability statement

The original contributions presented in the study are included in the article/Supplementary material, further inquiries can be directed to the corresponding author.

## Author contributions

All authors listed have made a substantial, direct, and intellectual contribution to the work and approved it for publication.

## Author disclaimer

The study presented was not performed or funded by the EPA and was not subject to the EPA's quality system requirements. The views expressed in this article are those of the author(s) and do not necessarily represent the views or policies of the U.S. Environmental Protection Agency. The United States Government has a royalty-free license throughout the world for all copyrightable material contained herein. Any opinions, findings, conclusions, or recommendations expressed in this material are those of the author(s) and do not necessarily reflect the views of the Office of Army Research. Any use of trade, firm, or product names is for descriptive purposes only and does not imply endorsement by the U.S. Government.
