# Supplementary material for: Identifying sources of antibiotic resistance genes in the environment using the microbial Find, Inform, and Test framework
Source: Front Microbiol. 2023 Sep 5;14:1223876. doi: 10.3389/fmicb.2023.1223876 (PMC10508347; doi:10.3389/fmicb.2023.1223876)
Supplement: Supplementary file 1 [file Data_Sheet_1.docx]

***Supplementary Material***

Identifying sources of antibiotic resistance genes in the environment using the microbial Find, Inform, and Test framework

**Authors**

Corinne Wiesner-Friedman^a^, Rachelle E. Beattie^b,d^, Jill R. Stewart^c^, Krassimira R. Hristova^d^, Marc L. Serre^c,*^

**Affiliations**

^a^Office of Research and Development, U.S. Environmental Protection Agency, 26 West Martin Luther King Drive, Cincinnati, Ohio 45268, United States

^b^ U.S. Geological Survey, Columbia Environmental Research Center, 4200 New Haven Road, Columbia, Missouri 65201, United States

^c^Gillings School of Global Public Health, Department of Environmental Sciences and Engineering, University of North Carolina at Chapel Hill, Chapel Hill, North Carolina, 27599, United States

^d^ Department of Biological Sciences, Marquette University, 1428 W Clybourn St, Milwaukee, Wisconsin 53233, United States

***Correspondence:** Corresponding Author: marc_serre@unc.edu

**Number of pages**

13

**Number of Figures**

1

1. Figure S1 Two examples of overarching databases that we used to represent sources for predicting how sources may impact ARG levels in riverbed sediments and surface water

**Number of Tables**

6

1. Table S1 Below detect counts from qPCR for ARGs out of the sample size
2. Table S2 All categories and candidate database options used in this study. All source categories are described in detail in Wiesner-Friedman *et al.*^1^
3. Table S3 Soil source categories and candidate database options as an extension of the potential sources and candidate spatial options outlined in Wiesner-Friedman *et al.*^1^
4. Table S4 Survey questions and responses from Wisconsin veterinarians
5. Table S5 Regression results for predicting the relative abundance of *erm(B), tet(W), qnrA, sul1, and inI1* (log10 gene*-*copies-per-16S-rRNA-copies) in sediment (columns toward the right) and surface water (left-most 5 columns). The sample size is indicated for each of the responses in each column. For each of the climatic and source terms the standardized regression coefficient, $\boldsymbol{\beta}$, is provided resulting from the *Test* stage of FIT. For each source term, there are two additional rows resulting from *Find* and *Inform* stages of the FIT framework. For each source term category (i.e., bovine or land-applied waste), the source description, the relative abundance ration (${RAR=10}^{\beta}) and$hyperparameters indicating the influence range around sources, $\boldsymbol{\alpha}$**,** are summarized. Precipitation term associations are shown in blue. The associations with freezing temperature are shown in white. Bovine source associations are in red. Land-applied waste sources are shown in yellow.
6. Table S6 Databases representing the selected source terms as a result of the *Find* stage of FIT.

Table of Contents

[S1 Details on the calculation of relative abundances and below detects 1](#_Toc114234248)

[S2 Details on source categories and candidate database options 1](#_Toc114234249)

[S3 Details on soil types 7](#_Toc114234250)

[S4 Details of the reliability score calculation 7](#_Toc114234251)

[S5 Details on the interpretation of standardized regression coefficients as RARs 8](#_Toc114234252)

[S6 Details on the veterinarian interviews conducted to understand antibiotic practices in dairy AFOs 8](#_Toc114234253)

[S6.1 Veterinarian interviews questions and methods 9](#_Toc114234254)

[S6.2 Synthesis of interview responses and discussion 10](#_Toc114234255)

[S7 Patterns of associations with recent and antecedent precipitation and freezing temperature 11](#_Toc114234256)

[S8 Details about the differences in databases selected for each response 14](#_Toc114234257)

[S9 Details on predicting source impacts at unsampled locations 15](#_Toc114234258)

# S1 Details on the calculation of relative abundances and below detects

ARGs and 16S rRNA were quantified from sediment and surface water. When quantification values were below the level of detection, the value was set to one-half of the limit of detection. Of the sampling dates (July 2016, September 2016, October 2016, February 2017, May 2017) most of the below detects occurred in July.

Table S1 shows the number of below detects out of the sample size. This demonstrates excellent detection rates in sediment and good detection rates in surface water.

**Table S1** Below detect counts from qPCR for ARGs out of the sample size

|  | *erm(B)* | *tet(W)* | *qnrA* | *sul1* | *intI1* |
| --- | --- | --- | --- | --- | --- |
| Riverbed Sediment | 2/90 | 9/90 | 0/90 | 0/90 | 1/90 |
| Surface Water | 12/98 | 17/98 | 2/98 | 11/98 | 15/98 |

There were not below detect values for 16S rRNA in surface water or sediment.

Once the below-detect values were set, the following equation was used to obtain the response data, expressed as a log10 relative abundance.

$\left\{ \begin{matrix} z_{i}=\frac{the absolute abundance of ARG (gene copies)}{16S rRNA abundance (gene copies)} \\ y_{i}=\log_{10} (z_{i}) \end{matrix} \right.$, (Eq. S1)

# S2 Details on source categories and candidate database options

Table S2 describes all the potential source categories and candidate database options used in this study. Most of these are described in detail in Wiesner-Friedman *et al.*^1^ However, we now add soil as a potential source and include a database obtained from the Wisconsin Department of Natural Resources. Table S3 summarizes the type of potential soil source, the different options available from different data classes (only one option was available per soil source), coding options, and databases, and provides a detailed description.

Maps showing the locations for each of databases of spatially distributed sources (u=1 through 9) can be found in Wiesner-Friedman *et al.*^2^ manuscript and supporting information.

**Table S2** All categories and candidate database options used in this study. All source categories are described in detail in Wiesner-Friedman *et al.*^1^

| Source Type*, u* | Potential Source  (Abbreviation) | Candidate Spatial Database |
| --- | --- | --- |
| 1 | **Animal Feeding Operations**  **(AFO)**  *See supporting information from Wiesner-Friedman et al.*^1^ *for depiction and descriptions of database AFO locations* | Option 1: County Database of Manure Storages (weighted) |
|  |  | Option 2: County Database of Manure Storages (unweighted) |
|  |  | Option 3: WPDES Database of CAFOs (unweighted) |
|  |  | Option 4: WPDES Database of CAFOs (weighted by animal units) |
| 2 | **Manure Application Fields**  **(ManureApp)**  *See supporting information from Wiesner-Friedman et al.*^1^ *for depiction and descriptions of database ManureApp locations* | Option 1: Land Cover Database of Crop Rotation |
|  |  | Option 2: Land Cover Database of Dairy Rotation |
| 3 | **Septic Systems**  **(Sep)**  *See supporting information from Wiesner-Friedman et al.*^1^ *for depiction and descriptions of database septic system locations* | Only option: County Database of Drainfield Locations (unweighted) |
| 4 | **Wastewater Treatment Plants**  **(WWTP)**  *See supporting information from Wiesner-Friedman et al.*^1^ *for depiction and descriptions of database WWTP locations* | Option 1: State Database of all WPDES wastewater treatment facilities |
|  |  | Option 2: State Database of WPDES Wastewater Treatment Facilities under the municipal category, weighted by population. |
| 5 | **All Land Applied Sludge Sites**  **(LAS)**  *See supporting information from Wiesner-Friedman et al.*^1^ *for depiction and descriptions of database of all land applied sludge site locations* | Option 1: State Database of WPDES Land Applied Sludge Sites weighted by acreage |
|  |  | Option 2: State Database of WPDES Land Applied Sludge sites (unweighted) |
| 6 | **Land Applied Sludge with Non-Industrial Waste**  **(DomLAS)**  *See supporting information from Wiesner-Friedman et al.*^1^ *for depiction and descriptions of database land applied sludge with non-industrial waste locations* | Option 1: State Database of WPDES Land Applied Sludge Sites with municipal or septage |
|  |  | Option 2: State Database of WPDES Land Applied Sludge Sites with septage |
|  |  | Option 3: State Database of WPDES Land Applied Sludge Sites with wastewater treatment |
| 7 | **Land Applied Sludge with Industrial Waste**  **(IndLAS)**  *See supporting information from Wiesner-Friedman et al.*^1^ *for depiction and descriptions of database land applied sludge with industrial waste locations* | Option 1: State Database of WPDES Land Applied Sludge Sites with Industrial Sludge or Wastewater |
|  |  | Option 2: State Database of WPDES Land Applied Sludge Sites with Industrial Wastewater |
|  |  | Option 3: State Database of WPDES Land Applied Sludge Sites from Food Processing |
| 8 | **High Intensity Developed Land Cover**  **(HID)**  *See supporting information from Wiesner-Friedman et al.*^1^ *for depiction and descriptions of database high intensity developed land cover locations* | Option 1: Land cover database of high intensity developed land cover as polygon |
|  |  | Option 2: Land cover database of high intensity developed land cover as gridded points |
| 9 | **Low Intensity Developed Land Cover**  **(LID)**  *See supporting information from Wiesner-Friedman et al.*^1^ *for depiction and descriptions of database low intensity developed land cover locations* | Option 1: Land cover database of low intensity developed land cover as polygon |
|  |  | Option 2: Land cover database of low intensity developed land cover as gridded points |
| 10 | **Soil Type A**  **(SA)**  *See Table* | Only option: Soil characteristics database where the dominant soil type is type A, which is the most pervious soil type and characterized by sand, sandy loam, loamy sand, or gravel. |
| 11 | **Soil Type C**  **(SC)** | Only option: Soil characteristics database where the dominant soil type is type C, which is the 2^nd^ most impervious soil type characterized by sandy clay loam. |
| 12 | **Soil Type D**  **(SD)** | Only option: Soil characteristics database where the dominant soil type is type D, which is the most impervious soil type and characterized by clay loam, silty clay loam, sandy clay, or silty clay. |

**Table S3** Soil source categories and candidate database options as an extension of the potential sources and candidate spatial options outlined in Wiesner-Friedman *et al.*^1^

| Source Type*, u* | Potential Source  (Abbreviation) | Candidate Spatial Database | Database description |
| --- | --- | --- | --- |
| Source Type*, u* | Potential Contaminant Source  (Abbreviation) | Candidate Spatial Database | Database description |
| 10 | **Soil Type A**  **(SA)** | Only option: Soil characteristics database where the dominant soil type is type A, which is the most pervious soil type and characterized by sand, sandy loam, loamy sand, or gravel. | This database of soil associations was created by the Wisconsin Department of Natural Resources (WDNR), the U.S. Geological Survey (USGS), the Wisconsin Geological & Natural History Survey (WGNHS), and the University of Wisconsin- Madison.  Coverage of this soil association was calculated as the percent of area within a 1 km radius around the sampling location with this soil type and then determining what the dominant soil type within that radius.  The soils in the United States are assigned to four groups (A, B, C, and D) and three dual classes (A/D, B/D, and C/D). ***The study area only has A, C, and D soil types.***  Group A. Soils having a high infiltration rate (low runoff potential) when thoroughly wet. These consist mainly of deep, well drained to excessively drained sands or gravelly sands. These soils have a high rate of water transmission.  <https://data-wi-dnr.opendata.arcgis.com/datasets/wi-dnr::gcsm-soil-characteristics/about> |
| 11 | **Soil Type C**  **(SC)** | Only option: Soil characteristics database where the dominant soil type is type C, which is the 2^nd^ most impervious soil type characterized by sandy clay loam. | Same as above  Group C. Soils having a slow infiltration rate when thoroughly wet. These consist chiefly of soils having a layer that impedes the downward movement of water or soils of moderately fine texture or fine texture. These soils have a slow rate of water transmission.  <https://data-wi-dnr.opendata.arcgis.com/datasets/wi-dnr::gcsm-soil-characteristics/about> |
| 12 | **Soil Type D**  **(SD)** | Only option: Soil characteristics database where the dominant soil type is type D, which is the most impervious soil type and characterized by clay loam, silty clay loam, sandy clay, or silty clay. | Same as above  Group D. Soils having a very slow infiltration rate (high runoff potential) when thoroughly wet. These consist chiefly of clays that have a high shrink-swell potential, soils that have a high water table, soils that have a claypan or clay layer at or near the surface, and soils that are shallow over nearly impervious material. These soils have a very slow rate of water transmission.  <https://data-wi-dnr.opendata.arcgis.com/datasets/wi-dnr::gcsm-soil-characteristics/about> |

# S3 Details on soil types

The soils in the United States are assigned to four groups (A, B, C, and D) and three dual classes (A/D, B/D, and C/D). The groups are defined as follows (see Table S3):

Group A. Soils having a high infiltration rate (low runoff potential) when thoroughly wet. These consist mainly of deep, well drained to excessively drained sands or gravelly sands. These soils have a high rate of water transmission.

Group B. Soils having a moderate infiltration rate when thoroughly wet. These consist chiefly of moderately deep or deep, moderately well drained or well drained soils that have moderately fine texture to moderately coarse texture. These soils have a moderate rate of water transmission.

Group C. Soils having a slow infiltration rate when thoroughly wet. These consist chiefly of soils having a layer that impedes the downward movement of water or soils of moderately fine texture or fine texture. These soils have a slow rate of water transmission.

Group D. Soils having a very slow infiltration rate (high runoff potential) when thoroughly wet. These consist chiefly of clays that have a high shrink-swell potential, soils that have a high water table, soils that have a claypan or clay layer at or near the surface, and soils that are shallow over nearly impervious material. These soils have a very slow rate of water transmission.

# S4 Details of the reliability score calculation

The details of this calculation are presented in the original manuscript and supporting information of the publication detailing the microbial Find, Inform, and Test Framework (<https://doi.org/10.1021/acs.est.1c01602>).^3^

# S5 Details on the interpretation of standardized regression coefficients as RARs

We can calculate an ${RAR}^{(u)} [i.e., z\left( s^{\left( u \right)}+1 \right)/z\left( s^{\left( u \right)} \right)={10}^{\beta_{u}}]$ from $\beta_{u}$which corresponds to the ratio of relative abundances from an increase of 1 unit in SPM (i.e. the relative abundance $z\left( s^{\left( u \right)}+1 \right)$ in the numerator is calculated for an SPM value $(s^{\left( u \right)}+1$) that is 1 unit larger than that of the denominator $z\left( s^{\left( u \right)} \right)$) .

# S6 Details on the veterinarian surveys conducted to understand antibiotic practices in dairy AFOs

## S6.1 Veterinarian survey questions and methods

An IRB was written for conducting interviews, and an exempt status was obtained due to anonymity and the study area encompassing a whole state. Emails were sent to veterinarian email addresses with the survey. Two phone interviews were conducted due to preference, and 2 surveys were completed online through Qualtrics. The survey is outlined in Table S4, and phone interviews were conducted in the same format as the survey, with room for more extended responses.

**Table S4** Survey questions and responses from Wisconsin veterinarians

| Question | Veterinarian #1 | Veterinarian #2 | Veterinarian #3 | Veterinarian #4 |
| --- | --- | --- | --- | --- |
| What antibiotic or antibiotics do you think is or are most frequently used on commercial/conventional dairy cattle operations and for what most common treatment or reason? | Beta lactams & ceftiofur for metritis & mastitis | Ceftiofur would be the most common antibiotic used in the adult dairy herd. It is used for the treatment of pneumonia, mastitis, metritis and footrot. In the young stock, ceftiofur is also used, but other common antibiotics are enrofloxacin, florfenicol, tulathromycin, and oxytetracycline used for the treatment of pneumonia. | Exenel (ceftiofur hydrochloride--cephalasporin), Excede (ceftiofur crystalline- cephalasporin), Polyflex (ampicillin), beta-lactams. Used for metritis, pneumonia, and foot problems. | ceftiofur |
| What antibiotic or antibiotics do you think is or are most frequently used on organic dairy cattle operations and for what most common treatment or reason? | None | I have no experience with organic dairies. | None | None |
| What antibiotic or antibiotics do you think is or are most frequently used on small family dairy farms and for what most common treatment or reason? | Beta lactams & ceftifur for metritis & mastitis | See above. | Small dairy farms are similar to conventional operation's use. |  |
| Have you seen any changes in the use of antibiotics in livestock over time? Describe to what these changes are attributed to? If no change has occurred, you can write "none" or explain further. | Less antibiotics are being used in general. More culturing to assure appropriate usage. | There has been a decreased use of antibiotics over time as dairy operations have improved their housing and management resulting in decreased cases of pneumonia, diarrhea and mastitis. | Over time, more antibiotics used to be used. A lot less usage of antibiotics for mastitis. Hardly sell antibiotics for mastitis anymore. There are better methods and products for testing and treatment now. "Flushing" with tetracyline used to be common practice. This vet does not like the "modern" way. "Flushing" would be used in the past 3 days after the cow passed the placenta to make sure the cow was "clean". Now the operations are supposed to monitor the cow's health after they have the calf to check for potential infection so that they would only use antibiotics if the cow was sick. However, this vet finds that the operations are not monitoring the cows well, so the placenta starts to rot and the cow becomes febrile. | None. |
| What do you think the prevalence of mastitis is among dairy cattle per 10,000? If you do not know, please write "not known". | 500 | Not known. | Not known. | Not known. |
| How long have you been working as a veterinarian with dairy cattle? | 1-5 years | 20+ years | 20+ years | 1-5 years |

## S6.2 Synthesis of interview responses and discussion

Out of the four veterinarians that responded, two of the veterinarians interviewed had greater than 20 years of experience in the field and indicated that antibiotic use has decreased over the years, especially for treatments of mastitis, of which they mentioned newer treatments, but did not identify them specifically. A common use of antibiotics is for treatment of infection from calving. One veterinarian discussed the use of antibiotics to prevent illness. “Tetracycline flushes” were described as when tetracyclines are administered to the calving mother for three days after calving to prevent infection. Currently guidelines have sought to decrease this practice, but veterinarians have noted that if operations are not monitoring cows well, the cow can “become febrile.” Due to ethical reasons, some veterinarians do not like the “modern way” because cows may be poorly monitored and can suffer. In the past, antibiotics were primarily used for the treatment of mastitis, but veterinarians indicated that newer treatments now exist. Our interviews suggest that veterinarians may be resilient to reducing antibiotic use if it leads to more animal suffering. Policies aimed at reducing the impacts of AFOs on antimicrobial resistance should work closely with veterinarians to identify other ways to reduce animal suffering.

According to the four veterinarians that we interviewed, Ceftiofur was the frequently prescribed antibiotic, which is a Beta-lactam.^4^ Other antibiotics that were identified as were enrofloxacin, florfenicol, tulathromycin, and oxytetracycline which belong to the broader classes of fluoroquinolones, sulfonamides, macrolides, and tetracyclines. This is important information due to barriers to sampling at dairy operations. The interviewed veterinarians demonstrate a wealth of information about dairy operations and are motivated by the well-being of animals. Policies aimed at reducing the impacts of AFOs on antimicrobial resistance should work closely with veterinarians to identify other ways to reduce animal suffering.

# S7 Patterns of associations with recent and antecedent precipitation and freezing temperature

The results from the *Find, Inform, and Test* (FIT) framework applied to the log10 antibiotic resistance gene relative abundance (log10 gene-copies-per-16S-rRNA-copies) for each of the five responses: *erm(B), tet(W), qnrA, sul1,* and *intI1* are shown in Table S5*.* Here, we focus on top part of the table corresponding to associations with precipitation and freezing temperature. We find that there are three patterns of association between recent precipitation and the effect of antecedent precipitation on recent precipitation: 1) wet antecedent conditions protect against contributions from a recent precipitation event (i.e., a one-standard-deviation increase in recent precipitation is associated with a ${|\beta}_{1}|$ *increase* in relative gene abundance, but a one-standard-deviation increase in antecedent precipitation *diminishes* that effect by ${|\beta}_{2}|$) 2) wet antecedent conditions exacerbate the contributing effects of a recent precipitation event (i.e., a one-standard-deviation increase in recent precipitation is associated with a ${|\beta}_{1}|$ *increase* in relative gene abundance, but a one-standard-deviation increase in antecedent precipitation *increases* that effect by ${|\beta}_{2}|$) and 3) recent precipitation decreases baseline levels of the gene relative abundance but this is diminished by wet antecedent conditions, which can overall increase levels (i.e., a one-standard-deviation increase in recent precipitation is associated with baseline ${|\beta}_{1}|$ *decrease* in relative gene abundance, but a one-standard-deviation increase in antecedent precipitation adds to that effect by ${|\beta}_{2}|$, which can lead to overall *increases*).

The log10 relative abundances of *erm(B), tet(W),* and *qnrA* (log10 gene-copies-per-16S-rRNA-copies) in surface water were characterized by the first pattern. For example, a one-standard-deviation increase in recent precipitation was associated with increases of 0.417 (p-value<0.05) increase in the log10 relative abundance of *erm(B)* in surface water*.* However, a one-standard-deviation increase in antecedent precipitation diminished the effect of recent precipitation by 0.383 (p-value<0.05). Similar to this pattern, we found that a one-standard-deviation increase in recent precipitation was associated with an increase of 0.637 (p-value<0.05) in the log10 relative abundance of *sul1*in surface water without any diminishing effect from antecedent precipitation.

The log10 relative abundances of *tet(W)* in sediment and *intI1* in surface water (log10 gene-copies-per-16S-rRNA-copies) were characterized by the second pattern. For example, a one-standard-deviation increase in recent precipitation was associated with an increase of 0.654 (p-value<0.05) in the log10 relative abundance of *tet(W)* in sediment. However, a one-standard-deviation increase in antecedent precipitation increased the effect of recent precipitation by 0.923 (p-value<0.05).

The log10 relative abundances of *erm(B)* and *intI1* in sediment (log10 gene-copies-per-16S-rRNA-copies) were characterized by the third pattern. For example, a one-standard-deviation increase in recent precipitation was associated with a decrease of 0.723 (p-value<0.05) in the log10 relative abundance of *erm(B).* However, the effect of this decrease was diminished by 0.561 (p-value<0.05) for a one-standard deviation increase in antecedent precipitation.

The first pattern describing the effect of antecedent precipitation on recent precipitation contributions only occurred for ARG responses in surface water. This is consistent with findings around the flush effect for fecal indicators and host-associated responses in surface water.^5–10^ For surface water, a second pattern was found for the relative abundance of *intI1.* This second pattern was also found for the *tet(W)* response in sediment. The third pattern only occurred for sediment responses and has not been reported before. Previously, wet events have been associated with elevated ARGs and ARG levels have been found to increase during and after precipitation events.^11–13^

Lastly, freezing temperature was not frequently selected by FIT across the 5 ARG responses in surface water and sediment (i.e., 10 responses). When selected for sediment, freezing temperature characterized decreases to the ARG responses. Freezing temperature was associated with decreases of 0.621 (p-value<0.10) and 0.812 (inclusion lowers AIC) in the log10 relative abundance of *erm(B)* and *intI1* in sediment. Freezing temperature was only selected once for surface water and characterized increases to the log10 relative abundance of *erm(B).* Freezing temperature was associated with a 1.93 (p-value<0.05) increase in the relative abundance of *erm(B)* in surface water. Overall, precipitation and freezing temperature were helpful to explaining between-sampling event variability, but studies are needed with a finer temporal resolution to untangle the associations between recent and antecedent precipitation on ARGs and ambient temperatures.

**Table S5** Regression results for predicting the relative abundance of *erm(B), tet(W), qnrA, sul1, and inI1* (log10 gene*-*copies-per-16S-rRNA-copies) in sediment (columns toward the right) and surface water (left-most 5 columns). The sample size is indicated for each of the responses in each column. For each of the climatic and source terms the standardized regression coefficient, $\boldsymbol{\beta}$, is provided resulting from the *Test* stage of FIT. For each source term, there are two additional rows resulting from *Find* and *Inform* stages of the FIT framework. For each source term category (i.e., bovine, land-applied waste, or soil), the source description, the relative abundance ration (${RAR=10}^{\beta}) and$hyperparameters indicating the influence range around sources, $\boldsymbol{\alpha}$**,** are summarized. Precipitation (Precip.) term associations are shown in blue. The associations with freezing temperature are shown in white. Bovine source associations are in red. Land-applied waste sources are shown in yellow.

| Environmental Matrix | | Riverbed Sediment | | | | | Surface Water | | | | |
| --- | --- | --- | --- | --- | --- | --- | --- | --- | --- | --- | --- |
| ARG  (*n*=sample size) | | ***erm(B)***  (*n*=91) | ***tet(W)***  (*n*=91) | | ***sul1***  (*n*=91) | ***intI1***  (*n*=91) | ***erm(B)***  (*n*=98) | ***tet(W)***  (*n*=98) | ***qnrA***  (*n*=98) | ***sul1***  (*n*=98) | ***intI1***  (*n*=98) |
| **Recent**  **Precip.** | Std. Regression  Coefficient  ($\boldsymbol{\beta}_{\boldsymbol{1}}\boldsymbol{)}$ | -0.723** | 0.654** | | *NS* | -0.330** | 0.415** | 0.635** | 0.213** | 0.637** | 0.277** |
| **Recent x Antecedent Precip.** | Std. Regression  Coefficient  ($\boldsymbol{\beta}_{\boldsymbol{2}}\boldsymbol{)}$ | 0.561** | 0.923** | | *NS* | 0.256** | -0.394** | -0.300** | -1.10** | *NS* | 0.637** |
| **Freezing** | Regression  Coefficient  ($\boldsymbol{\beta}_{\boldsymbol{3}}\boldsymbol{)}$ | -0.621* | *NS* | | *NS* | -0.801** | 1.94** | *NS* | *NS* | *NS* | *NS* |
| **Bovine Sources** | Bovine Source  Description | GORF  AFO  (via ground hauling of manure to application fields) | GORF  AFO  (via ground hauling of manure to application fields) | | *NS* | *NS* | ORF  AFO  (via ground hauling of manure to application fields) | ORF  AFO | ORF  AFO | ORF  Manure App. Fields | ORF  Manure App. Fields |
|  | Std. Regression  Coefficient  ($\boldsymbol{\beta}_{\boldsymbol{u}}\boldsymbol{)}$ | 0.199* | 0.303* | | *NS* | *NS* | 0.162** | 0.247** | 0.173** | 0.148* | 0.134 |
|  | RAR  ($\boldsymbol{10}^{\boldsymbol{\beta}_{\boldsymbol{u}}}$) | 1.58* | 2.01* | | *NS* | *NS* | 1.45** | 1.77** | 1.49** | 1.41* | 1.36 |
|  | Influence Range  ($\boldsymbol{\alpha}_{\boldsymbol{O}})$ | $<10 \mathrm{km}$ | | | | | | | | | |
| **Land-App. Waste Sources** | Land applied waste Source  Description | *NS* | *NS* | | Land applied waste- *residential* | Septage ground transport to land app. sludge- *residential* | *NS* | Land applied waste- *industrial* | *NS* | *NS* | Land applied waste- *industrial* |
|  | Std. Regression  Coefficient  ($\boldsymbol{\beta}_{\boldsymbol{u}}\boldsymbol{)}$ | *NS* | *NS* | | 0.211** | 0.155* | *NS* | 0.134* | *NS* | *NS* | 0.148* |
|  | RAR  ($\boldsymbol{10}^{\boldsymbol{\beta}_{\boldsymbol{u}}}$) | *NS* | *NS* | | 1.63** | 1.43* | *NS* | 1.36* | *NS* | *NS* | 1.41* |
|  | Influence Range  ($\boldsymbol{\alpha}_{\boldsymbol{O}})$ | $<10 \mathrm{km}$ | | | | | | | | | |
| **Soil Sources** | Soil Source Description | *NS* | | *NS* | Type A (Sand, Sandy loam, Loamy sand, Gravel) | Type A (Sand, Sandy loam, Loamy sand, Gravel) | Type D  (Clay loam, silty clay loam, sandy clay, silty clay) | *NS* | *NS* | *NS* | *NS* |
|  | Regression  Coefficient  ($\boldsymbol{\beta}_{\boldsymbol{u}}\boldsymbol{)}$ | *NS* | | *NS* | 0.402** | 0.253 | 0.231 | *NS* | *NS* | *NS* | *NS* |
|  | RAR  ($\boldsymbol{10}^{\boldsymbol{\beta}_{\boldsymbol{u}}}$) | *NS* | | *NS* | 2.52** | 1.79 | 1.70 | *NS* | *NS* | *NS* | *NS* |
|  | Influence Range  ($\boldsymbol{\alpha}_{\boldsymbol{Radius}})$ | 1 km | | | | | | | | | |

**indicates p-value<0.05, * indicates p-value<0.10

NS indicates that no terms were selected for the source category.

Other source categories not selected: Wastewater treatment plants, septic systems, and developed land cover

No terms were selected by AIC for the log10 relative abundance of *qnrA* in sediment

# S8 Details about the differences in databases selected for each response

After exploring many databases representing bovine sources or land-applied waste sources of elevated AMR, we found that databases were differently characterized between sediment and surface water responses (see Table S6). For sediment, AFO contributions were represented by WPDES CAFOs via hauling to manure application fields (i.e., crop rotation or dairy rotation land cover). Whereas for surface water, AFO contributions were represented by their manure storages overtook other bovine source terms. Previously, a similar pattern was revealed for bovine-associated fecal contamination from these samples.^2^ For land-applied waste contributions, residential categories were associated with sediment ARG relative abundance, whereas industrial categories were associated with surface water ARG relative abundance. These differences may reflect different overland pathways of contamination to sediment or surface water, different detection attributes in sediment versus surface water, or this may reflect the higher impact of some source locations on ARG levels in rivers during flooding conditions, when surface water was able to be sampled, but not sediment. Future work examining differences between surface water and sediment should consider accessibility and approaches for sampling sediment during flooding conditions. Sampling during many different flow events and at a finer temporal resolution than was possible in this work can help to better understand how precipitation and flooding conditions may impact the dissemination of microbial pollution from sources.

**Table S6** Databases representing the selected source terms as a result of the *Find* stage of FIT.

|  | Sediment | | | | Surface Water | | | | |
| --- | --- | --- | --- | --- | --- | --- | --- | --- | --- |
|  | ermB | tetW | sul1 | intI1 | ermB | tetW | qnrA | sul1 | intI1 |
| Source Terms |  | | | | | | | | |
| Bovine Sources  **Database(s) representing spatially distributed sources** | AFO  (via ground transport of manure to application fields)  ***From:* WPDES CAFO weighted by Animal Units**  ***To:* Crop rotation** | AFO  (via ground transport of manure to application fields)  ***From:* WPDES CAFO weighted by Animal Units**  ***To:* Dairy rotation** | AFO  (via ground transport of manure to application fields)  ***From:* WPDES CAFO (unweighted)**  ***To:* Crop rotation** | NS | AFO  (via ground transport of manure to application fields)  ***From:* Remotely sensed manure storages (unweighted)**  ***To:* Crop rotation** | AFO  **Remotely sensed manure storages weighted by log-total-gallons of manure** | AFO  **Remotely sensed manure storages weighted by log-total-gallons of manure** | Manure App. Fields  **Crop rot. land cover** | Manure App. Fields  **Crop rotation land cover** |
| Land Application of Sludge  **Database(s) representing spatially distributed sources** | NS | NS | Land Applied Sludge- Residential  **WPDES Land applied sludge from treated septage or municipal wastewater** | Septage ground transport to land application sites  ***From:* Septic Systems**  ***To:* WPDES Land applied sludge from treated septage or municipal wastewater** | NS | Land Applied Sludge- Industrial  **WPDES Land applied sludge from treated food processing** | NS | NS | Land Applied Sludge- Industrial  **WPDES Land applied sludge from treated industrial sludge** |

# S9 Details on predicting source impacts at unsampled locations

In the main paper there are depictions provided of the estimated source contributions from AFOs via manure application to sediment ARG relative abundance and industrial land-applied waste to surface water ARG relative abundance based on upper bounds of the hyperparameters obtained from FIT and using overarching classes of databases (see Figure S2) from those selected from FIT. These spatial source locations, all river network associations, associated weighting for sources, and hyperparameters values were input into the GORF spatial predictor model to define contributions from AFOs via manure application and the ORF spatial predictor model to define contributions from industrial land-applied waste. However, rather than z-scoring, the GORF and ORF were standardized based on the non-standardized values at our studies sampling sites and events (i.e., the values divided by the standard deviation of the values at sampling sites).

crop rotation land cover

dairy rotation land cover

cash grain

Continuous corn

Etc.

industrial land-applied waste

industrial wastewater

industrial sludge

food processing

**Figure S1** Two examples of overarching databases that we used to represent sources for predicting how sources may impact ARG levels in riverbed sediments and surface water

**Funding Sources**

This work was supported by a grant from the National Institute of Environmental Health Sciences (NIEHS) T32ES007018. This research was funded in part by the Marquette University Innovation Grant, NSF grant 1316318 as part of the joint NSF-NIH-USDA Ecology and Evolution of Infectious Diseases program, the Engineering Research Centers Program of the National Science Foundation under NSF Cooperative Agreement No. EEC-2133504, and the Department of Army award W9132T2220001 issued by the Office of Army Research.

**Acknowledgements**

The research presented was not performed or funded by EPA and was not subject to EPA’s quality system requirements. The views expressed in this article are those of the author(s) and do not necessarily represent the views or the policies of the U.S. Environmental Protection Agency. The United States Government has a royalty-free license throughout the world in all copyrightable material contained herein.  Any opinions, findings, and conclusions or recommendations expressed in this material are those of the author(s) and do not necessarily reflect the views of the Office of Army Research. Any use of trade, firm, or product names is for descriptive purposes only and does not imply endorsement by the U.S. Government.

Bibliography

(1) Wiesner-Friedman, C.; Beattie, R. E.; Stewart, J. R.; Hristova, K. R.; Serre, M. L. Characterizing Differences in Sources of and Contributions to Fecal Contamination of Sediment and Surface Water with the Microbial FIT Framework. *Environ. Sci. Technol.* **2022**, *56*, 4231–4240.

(3) Wiesner-Friedman, C.; Beattie, R. E.; Stewart, J. R.; Hristova, K. R.; Serre, M. L. Microbial find, inform, and test model for identifying spatially distributed contamination sources: framework foundation and demonstration of ruminant bacteroides abundance in river sediments. *Environ. Sci. Technol.* **2021**, *55*, 10451–10461.

(4) Dowling, P. M. Pharmacologic Principles. In *Equine Internal Medicine*; Elsevier, 2004; pp. 169–233.

(5) Riedel, T. E.; Thulsiraj, V.; Zimmer-Faust, A. G.; Dagit, R.; Krug, J.; Hanley, K. T.; Adamek, K.; Ebentier, D. L.; Torres, R.; Cobian, U.; et al. Long-term monitoring of molecular markers can distinguish different seasonal patterns of fecal indicating bacteria sources. *Water Res.* **2015**, *71*, 227–243.

(6) BERTRAND-KRAJEWSKI, J.-L.; CHEBBO, G.; SAGET, A. DISTRIBUTION OF POLLUTANT MASS VS VOLUME IN STORMWATER DISCHARGES AND THE FIRST FLUSH PHENOMENON. *Water Res.* **1996**.

(7) Sansalone, J. J.; Cristina, C. M. First flush concepts for suspended and dissolved solids in small impervious watersheds. *J. Environ. Eng.* **2004**, *130*, 1301–1314.

(8) Stumpf, C. H.; Piehler, M. F.; Thompson, S.; Noble, R. T. Loading of fecal indicator bacteria in North Carolina tidal creek headwaters: hydrographic patterns and terrestrial runoff relationships. *Water Res.* **2010**, *44*, 4704–4715.

(9) Holcomb, D. A.; Messier, K. P.; Serre, M. L.; Rowny, J. G.; Stewart, J. R. Geostatistical prediction of microbial water quality throughout a stream network using meteorology, land cover, and spatiotemporal autocorrelation. *Environ. Sci. Technol.* **2018**, *52*, 7775–7784.

(10) Coulliette, A. D.; Money, E. S.; Serre, M. L.; Noble, R. T. Space/time analysis of fecal pollution and rainfall in an eastern north carolina estuary. *Environ. Sci. Technol.* **2009**, *43*, 3728–3735.

(11) Barkovskii, A. L.; Babb, C. M.; Hurley, D.; Shin, E. Origins and environmental mobility of antibiotic resistance genes, virulence factors and bacteria in a tidal creek’s watershed. *J. Appl. Microbiol.* **2015**, *118*, 764–776.

(12) Ahmed, W.; Zhang, Q.; Lobos, A.; Senkbeil, J.; Sadowsky, M. J.; Harwood, V. J.; Saeidi, N.; Marinoni, O.; Ishii, S. Precipitation influences pathogenic bacteria and antibiotic resistance gene abundance in storm drain outfalls in coastal sub-tropical waters. *Environ. Int.* **2018**, *116*, 308–318.

(13) Jang, J.; Kim, M.; Baek, S.; Shin, J.; Shin, J.; Shin, S. G.; Kim, Y. M.; Cho, K. H. Hydrometeorological Influence on Antibiotic-Resistance Genes (ARGs) and Bacterial Community at a Recreational Beach in Korea. *J. Hazard. Mater.* **2021**, *403*, 123599.
